# Healthy Kai (Food) Checker Web-Based Tool to Support Healthy Food Policy Implementation: Development and Usability Study

**DOI:** 10.2196/60447

**Published:** 2025-01-13

**Authors:** Magda Rosin, Cliona Ni Mhurchu, Elaine Umali, Sally Mackay

**Affiliations:** 1 Department of Epidemiology and Biostatistics School of Population Health, Faculty of Medical and Health Sciences University of Auckland Auckland New Zealand; 2 Centre for Translational Health Research: Informing Policy and Practice (TRANSFORM) Faculty of Medical and Health Sciences University of Auckland Auckland New Zealand; 3 National Institute for Health Innovation, School of Population Health, Faculty of Medical and Health Sciences University of Auckland Auckland New Zealand

**Keywords:** healthy food policy, policy implementation, nutrition, food environment, hospital, workplace, web-based tool, digital tool, database, user testing, food, drink, nutrition professionals, acceptability, usability

## Abstract

**Background:**

Public health programs and policies can positively influence food environments. In 2016, a voluntary National Healthy Food and Drink Policy was released in New Zealand to improve the healthiness of food and drinks for hospital staff and visitors. However, no resources were developed to support policy implementation.

**Objective:**

This study aimed to design, develop, and test a new web-based tool to support food providers implementing the National Healthy Food and Drink Policy in New Zealand.

**Methods:**

The Double Diamond model, a structured framework with 4 design phases, was used to design and develop a web-based tool. Findings from our previous research, such as (1) systematic review of barriers and facilitators to workplace healthy food policy implementation; (2) scoping review of current tools and resources available in New Zealand, Australia, and Canada; (3) interviews with food providers and public health nutrition professionals; and (4) food and drink availability audit results in New Zealand hospitals were used in the “Discover” (understanding of current gaps) and “Define” (prioritizing functions and features) phases. Subsequent phases focused on generating ideas, creating prototypes, and testing a new web-based tool using Figma, a prototyping tool. During the “Develop” phase, project stakeholders (11 public health nutrition professionals) provided feedback on the basic content outline of the initial low-fidelity prototype. In the final “Deliver” phase, a high-fidelity prototype resembling the appearance and functionality of the final tool was tested with 3 end users (public health nutrition professionals) through interactive interviews, and user suggestions were incorporated to improve the tool.

**Results:**

A new digital tool, Healthy Kai (Food) Checker—a searchable database of packaged food and drink products that classifies items according to the Policy’s nutritional criteria—was identified as a key tool to support Policy implementation. Of 18 potential functions and features, 11 were prioritized by the study team, including basic and advanced searches for products, sorting list options, the ability to compile a list of selected products, a means to report products missing from the database, and ability to use on different devices. Feedback from interview participants was that the tool was easy to use, was logical to navigate, and had an appealing color scheme. Suggested visual and usability improvements included ensuring that images represented the diverse New Zealand population, reducing unnecessary clickable elements, adding information about the free registration option, and including more frequently asked questions.

**Conclusions:**

Comprehensive research informed the development of a new digital tool to support implementation of the National Healthy Food and Drink Policy. Testing with end users identified features that would further enhance the tool’s acceptability and usability. Incorporation of more functions and extending the database to include products classified according to the healthy school lunches program policy in the same database would increase the tool’s utility.

## Introduction

### Background

Despite the importance of healthy eating in the prevention of chronic diseases, the nutritional quality of diets worldwide does not align with evidence-based dietary guidelines, with widespread overconsumption of unhealthy and ultraprocessed foods and drinks [1,2]. However, foods are chosen in the context of local food environments [3,4], which in New Zealand are dominated by less healthy choices [5]. Workplace food environments, including hospitals, have been recognized as an important factor for employees’ health promotion and well-being due to their potential for broad and sustainable population reach [6], as well as the fact that the majority of the adult population works [7-9]. When this study began, the New Zealand health system was structured into 20 district health boards (DHBs) responsible for delivering national health care services through hospitals and clinical centers. However, in 2022, the DHB system was disestablished as part of a national health system reform [10]. The provision of health care services was centralized under the newly established Health New Zealand—Te Whatu Ora agency that directly employs around 90,000 staff, making it a major nationwide employer [11].

A voluntary National Healthy Food and Drink Policy (the Policy) was released in New Zealand in 2016 [12] to encourage hospitals to provide healthier food and drink options for staff and visitors (not inpatients). The Policy includes a traffic light classification system for food and drinks, where *healthy* (*Green*) options should make up at least 55% of available items; the *less healthy* (*Amber*) options that might still provide some nutritive value should be less prevalent (<45% of available items); and the *unhealthy* (*Red*) items, such as confectionery, deep-fried foods and sugar-sweetened drinks, are not permitted [12]. Yet, 2021 evaluation findings showed that 5 years after the introduction of the voluntary Policy in 2016, only 8 of the 20 DHBs had adopted the Policy, resulting in regional inconsistency [13]. Furthermore, on-site audits of food and drink availability in 2021/2022 indicated that the implementation was inconsistent and largely unsuccessful, with *Red* and *Amber* choices predominating [14].

### Previous Work

We conducted a systematic literature review of facilitators and barriers to implementing healthy food and drink policies in public sector workplaces. The review indicated that tailored supportive tools and resources facilitate the implementation of policies promoting healthy food environments [15]. A key reason why such support is required is because identifying healthier items can be challenging for food providers, who may find the policy’s nutrition criteria too complex to understand without nutrition expertise [15]. The supportive tools identified in the systematic review [15] were closely aligned with the supporting materials recommended by the World Health Organization [16] in their 2021 action framework for the implementation of effective healthy food policies in public settings. Examples of useful tools included administrative materials (eg, contract templates and standardized procedural guidelines); customer communication resources, compliance monitoring tools; and databases of compliant recipes, suppliers, and products [15,16]. The most useful tools were policy specific, readily available, and centrally provided, limiting the burden on individual food providers [15]. Importantly, supportive tools needed to be practical, up-to-date, user-friendly, and useful to those implementing a healthy food policy [15].

As part of a scoping review, we identified and evaluated the currently available tools used in public sector workplace policy implementation in New Zealand, Australia, and Canada [17]. Just 2 paper-based tools were related to the New Zealand Policy: guidelines to make better pies [18] and an event and fundraiser guide aimed at schools and community groups [19], and they were likely insufficient to facilitate implementation of the Policy [17]. No other tools were developed to support the New Zealand Policy implementation in contrast to most Australian food environment policies. Nevertheless, the scoping review [17] provided information on the features, usability, and quality of the available tools, which can be used to guide the development of new tools or update of existing tools. For example, for digital tools, which were less common than paper-based tools, logical navigation, clear search and result structure, consistent style, and unambiguous indication of products’ compliance in a web-based database were perceived as favorable features [17].

To identify which implementation tools were needed in New Zealand, we interviewed hospital food providers and public health nutrition professionals who were members of the National Food and Drink Environments Network (the Network) supporting implementation of the Policy in their respective DHBs [20]. Apart from customer communication materials, which are in development by Health New Zealand (the Network virtual meetings, 2023), interview participants suggested 2 digital tools that could support the New Zealand Policy implementation: a food and drink audit tool and an up-to-date database showing the traffic light classification of packaged products. Our research team previously developed a digital monitoring tool [21] with an embedded traffic light classification algorithm to systematically conduct the on-site audits of food and drinks available in New Zealand hospitals [14]. The second tool, a web-based product database, was requested because food providers and public health nutrition professionals spent significant time looking for products on the web and manually assessing individual products against the Policy criteria [20]. Furthermore, there has been no platform for food manufacturers and suppliers to communicate with the hospital food providers about food and drink products that may comply with the Policy [20].

### Rationale and Objective

Usability and appearance are likely to impact the usage of web-based tools. To develop the most optimal and feasible tool that meets the needs of its end users, user experience and user interface (UX/UI) design principles and user testing are needed [22]. A user-centered design approach is commonly used in fields that develop and use digital tools such as health care management [23], grocery shopping websites [22], and physical activity apps [24]. However, there is limited research on developing and testing tools designed to support the implementation of healthy food and drink policies.

The tool development was a component of the Healthy Policy Evaluation (HYPE) study that comprehensively evaluated the implementation of the National Healthy Food and Drink Policy in New Zealand and determined which tools and resources were needed to support Policy implementation. This paper aims to describe the development and user testing of a new prototype web-based tool to facilitate classification of packaged products according to the National Healthy Food and Drink Policy traffic light criteria.

## Methods

### Overview

**Figure 1:**
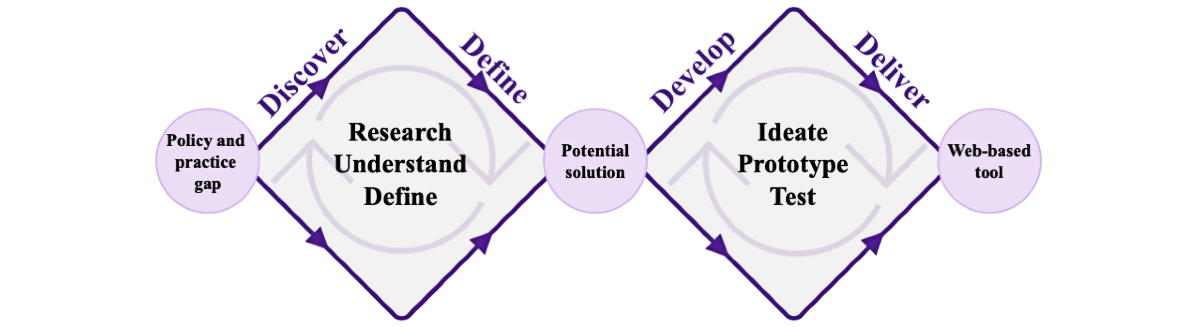
The Double Diamond model, illustrating the 4 phases (Discover, Define, Develop, and Deliver) used to guide the development of the Healthy Kai (Food) Checker web-based tool. Arrows pointing toward the tips of the diamonds demonstrate the diverging phases of the process characterized by creative thinking and the expansion and exploration of different ideas and possibilities. Arrows pointing away from the tips of the diamonds reflect the converging phases of the process where progressive decision-making occurs by focusing, prioritizing, and narrowing down the ideas and possibilities toward the most feasible and relevant solution. Circular arrows within the diamonds illustrate the iterative nature of the design process (adapted from the UK Design Council’s Double Diamond model [30] under Creative Common License CC BY 4.0 [52]).

This research was underpinned by pragmatism as the methodological paradigm [25-27]. We focused on practical and carefully considered decisions to develop the most effective and efficient prototype tool to facilitate implementation of the New Zealand Policy by incorporating a user-centered approach that emphasized the needs and preferences of product end users (hospital food providers and Network members [public health nutrition professionals]) [28,29]. The design and development of the new tool were guided by the Double Diamond model (Figure 1) created by the UK Design Council [30]. The model outlines an iterative and flexible process of identifying a real-world problem or gap, defining a possible solution, and finally developing a tangible, valuable, and well-informed outcome addressing the identified problem or gap [30]. The framework is not linear or rigid, and the phases and diamonds can overlap to produce the most optimal tool [30,31] and represent the evolving innovation process and continually changing digital space [30]. The Double Diamond model has been revised over the years by integrating additional steps within the framework, although the principles and the 4 major phases (Discover, Define, Develop, and Deliver) remain largely unchanged [32]. The Double Diamond phases were completed with the assistance of a subcontracted UX/UI designer, and study authors reviewed and provided feedback on all design and development outputs to ensure accurate representation and alignment with end user needs and the aims and scope of the Policy.

The UX/UI design approach could be broadly defined as “the process of designing physical or digital products that are useful, easy to use, and provide a great experience in interacting with them” [28]. Key UX/UI design principles [28,29,34] applied in this study were as follows:

1. User-centered design: designing and developing focused on the needs and preferences of end users (prioritizing accessibility and usability) and collaborating and seeking feedback from key stakeholders throughout the process.
2. Clear information hierarchy: arranging and presenting information in the web-based tool in a logical and organized way to ensure easy and user-friendly navigation and understanding of the content.
3. Visual and interaction consistency: maintaining a consistent and harmonious visual style to achieve a cohesive and professional look and ensuring predictable and consistent performance of the same style elements.
4. Feedback and confirmation: providing instant feedback and confirmation messages on successful user actions and warnings to prevent user errors (eg, deletion).
5. Mobile responsiveness: creating a seamless experience on both desktop and mobile platforms by optimizing the design to adapt to various devices.

### Ethical Considerations

Ethical approval for the HYPE study was granted by the Auckland Health Research Ethics Committee (reference AH2519). Subsequent locality approvals were sought from individual DHBs. All usability testing participants provided written consent to participate in the virtual interactive interviews. We approached potential participants up to 3 times by email between May and August 2023, explaining the purpose of the study and providing a detailed participant information sheet explaining the testing procedure. Participants who agreed to take part were sent a consent form, which they signed and returned by email. Participants had the option to have their *whānau* (family) present during the interview and were reimbursed for their time with a New Zealand $50 (US $30) grocery voucher. Study data were deidentified to ensure confidentiality and privacy.

### Discover Phase

In this phase, we compiled findings from other components of the HYPE study outlined in the *Introduction* section [14,15,17,20] relevant to designing and developing the new tool. The findings included thematic analysis codes generated during the coding of studies in the systematic review [15] and interview data [20], as well as the features, usability, and quality of the tools assessed in the scoping review [17]. Furthermore, we obtained screenshots of the digital tools included in the scoping review [17] and the websites of the major food suppliers, distributors, and retailers in New Zealand. We collated the findings using the whiteboard function in the web-based Miro software tool (RealtimeBoard, Inc).

### Define Phase

In the second phase, we analyzed and refined findings and outputs compiled in the Discover phase to define a feature and function requirement blueprint for the new tool. First, we created a user persona of a fictional end user; defined challenges they might face (pain points); and described their needs, actions they want to take, and expected benefits of using the new tool (a user story) [28,34]. Second, we further reviewed similar available web-based tools [17] in Australia [35,36] and Canada [37] and compared them on their UX/UI aspects of “first impressions,” “website visual design” features, and “website interaction” (features, accessibility, user flow, and navigation) [28,29,34].

In the next step, we used the Must have, Should have, Could have, Won’t have (MoSCoW) prioritization framework to define features and functions that were deemed “Must have” (essential and critical), “Should have” (important but not critical), “Could have” (desirable but not critical), and “Won’t have” (out of scope and excluded in the current design but could be considered in the future) [38]. We considered features identified under the “Must have” and “Should have” as core elements to be incorporated into the new prototype web-based tool.

In the final step, we focused on understanding and carefully planning the path a user would take when interacting with the web-based tool from the initial starting point (eg, home page) to the final step (eg, finding suitable products and checking their classification) [28,34]. The user flow diagram was an important starting point for creating an efficient and easy-to-use web-based tool [39]. The corresponding site map outlined the hierarchical structure of the tool’s pages, indicating how pages are linked, grouped, and prioritized in the structural tree [40], and we progressively simplified it to essential pages that aligned with the user flow diagram content.

### Develop Phase

We used Figma, a prototyping and design tool, for the remaining Develop and Deliver phases. In the Develop phase, we created a wide range of ideas for the prototype web-based tool underpinned by the outputs from the Define phase and the UX/UI principles outlined earlier. We started with wireframes, a basic arrangement of text, images, and buttons on the web pages before any additional visual elements or content were included [28].

The subsequent design included low-fidelity prototypes containing more details than wireframes, allowing us to test and refine ideas and concepts [28,41]. We engaged with the Network (n=36 members, consisting of public health professionals and dietitians representing their respective DHBs, as well as nutrition and public health advisors from the Ministry of Health and New Zealand Heart Foundation) as key project stakeholders and asked for their feedback and input on the low-fidelity prototypes before committing to a specific design. The Network was provided with a document via email containing different design options alongside a small set of questions about their preferred features, structure, layout, and content (Multimedia Appendix 1). Feedback was collated during the subsequent monthly Network virtual meeting (11 members were present) and used to refine the prototype further.

### Deliver Phase

#### Overview

In the Deliver phase, we developed a comprehensive high-fidelity prototype with interactive and functional elements, such as clickable content, buttons, and menus [28,41]. The tool’s pages were linked, mirroring the navigational flow of a live website and providing a realistic user experience, which allowed us to test user interaction with the prototype and refine the user journey [28,41]. We tested the prototype multiple times and once with a colleague to further improve and refine the high-fidelity prototype before testing it with end users.

#### Interactive Interviews to Test Usability of the Prototype Web-Based Tool

We conducted usability testing that involved interactive virtual interviews in which user interaction and engagement with the prototype were evaluated to determine user preferences and behaviors related to the prototype tool [42]. The testing also identified UX/UI challenges and pain points that needed addressing or redesigning to improve the tool [42].

#### Participant Selection and Recruitment

Potential participants (end users) were food providers (for staff and visitors) and members of the Network (public health nutrition professionals) within any of the New Zealand hospitals who had previously participated in the stakeholder interviews (n=12) [20] and who expressed willingness to test the new web-based tool, as well as other key stakeholders who had previously used the Policy and were familiar with its traffic light classification system.

#### Data Collection

The usability test interview combined 2 methods: performance testing [43], in which participants were given tasks to complete while interacting with the prototype, and visual design testing [44], in which participants were asked to evaluate visual aspects of the prototype tool. The usability interview guide (Multimedia Appendix 2) included questions about the prototype tool’s engagement, intuitive usage, functionality, navigation, quality, and user-friendliness, as well as scenarios and tasks related to the tool’s functions.

At the beginning of the virtual interviews, we explained the research purpose (testing the usability and user experience of the design product and not the participants’ ability to use the prototype), process, and participants’ rights; answered any questions; and verbally confirmed the permission to video record the interviews (as previously agreed to on the consent form). We provided participants with a digital link to access the Figma prototype and asked them to share their screen to demonstrate their interaction with the prototype’s features and functions. Participants were encouraged to think aloud during testing and to comment on the design, their preferences, and how the tool could be improved. All interviews were transcribed verbatim, and participants were given the opportunity to review and edit their transcripts.

#### Data Analysis

One author watched interview recordings multiple times and reviewed transcripts to analyze how participants interacted with the tool; how intuitive, engaging, efficient, and accessible the prototype tool was; and what changes were needed to improve the user experience. Participants’ suggestions, comments, and interactions were recorded on the printouts of the prototype. The results were shared and discussed with the UX/UI designer, who incorporated the feedback to improve and refine the design of the prototype. Textbox 1 summarizes the methods used and outputs in the phases of the Double Diamond model.

**Phases and outputs of the Double Diamond model used in the design and development of the new prototype Healthy Kai (Food) Checker web-based tool to support implementation of the National Healthy Food and Drink Policy in New Zealand hospitals.**

***Discover—divergent research phase* (understanding user needs and challenges they face, exploring similar tools available)**

- A systematic review [15] and scoping review findings [17] about tools and resources used internationally in implementation of healthy food and drink policies.
- Healthy Policy Evaluation (HYPE) study interview findings [20] specific to the characteristics and needs of the target audience (food providers and Network members [public health nutrition professionals]).
- HYPE study food and drinks audit findings [14], including types of products available.
- Layout and structure characteristics of existing digital tools [17] and major retailer and supplier websites.

***Define—convergent synthesis phase* (analyzing and refining insights generated in the Discover phase)**

- User persona of a fictional food provider, including their pain points and user story.
- Review and comparison of the user experience and user interface features of similar tools [35-37].
- Requirements blueprint for the new tool (refined by considering project timelines and the available budget).
- New tool features prioritized with the Must have, Should have, Could have, Won’t have (MoSCoW) framework [38].
- Visual user flow diagram (outlining actions, decisions, and steps users take through the tool).
- Structural site map of the pages in the tool.

***Develop—divergent ideation phase* (creating basic outlines of the prototype interface)**

- Wireframes, a basic skeletal outline of the website interface and elements.
- Low-fidelity prototype (a simple, monotone, and static representation of a prototype).
- Informal feedback from project stakeholders on the low-fidelity prototype.

***Deliver—convergent implementation phase* (refining of the prototype and usability testing)**

- High-fidelity prototype (detailed, interactive, and realistic representation closely resembling the final web-based tool).
- Interactive usability testing with end users.

## Results

### Define Phase

**Figure 2:**
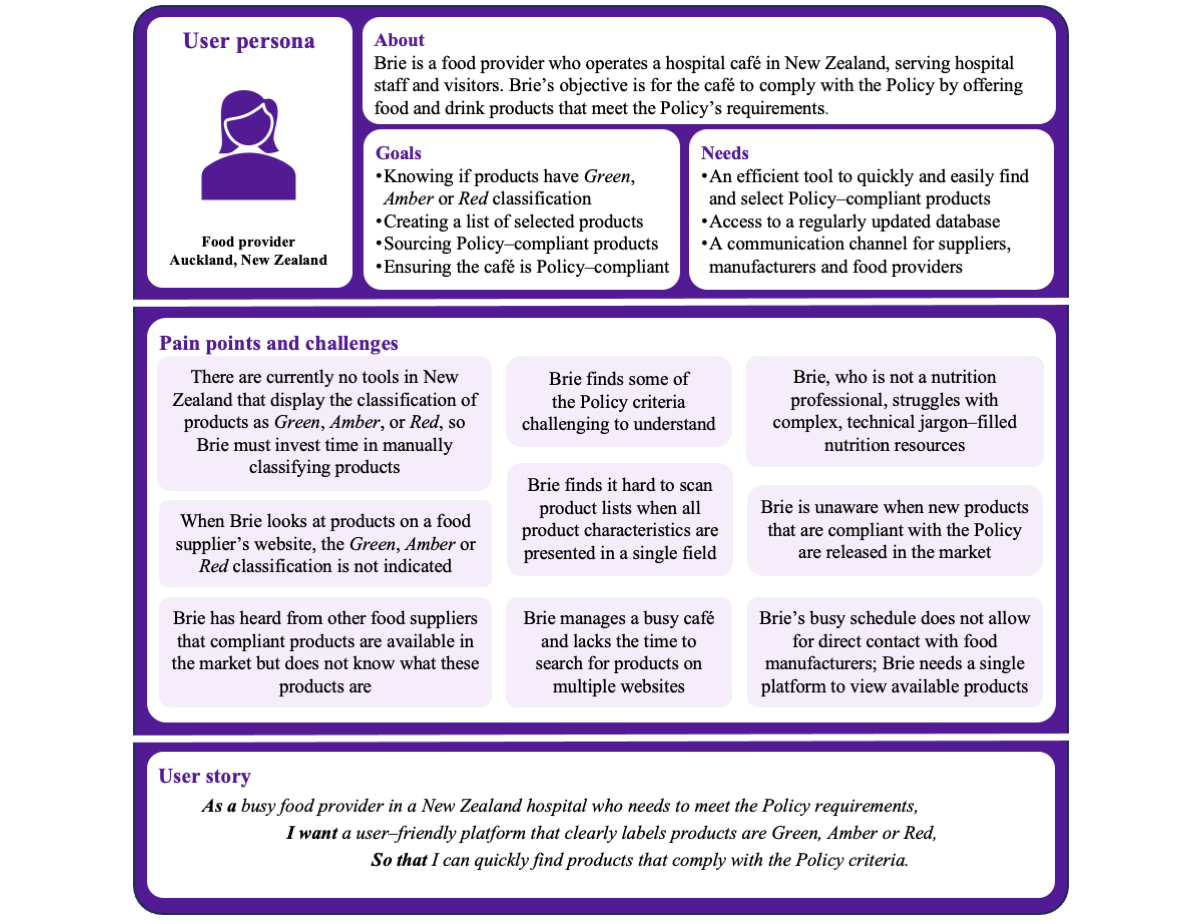
User persona, pain points, and user story of a fictional hospital food provider in New Zealand. The figure illustrates the characteristics, challenges, and narrative of a typical hospital food provider, highlighting their needs and experiences in implementing the National Healthy Food and Drink Policy and expected benefits of using the new tool.

The user persona of a fictional food provider, including their characteristics, challenges, and user story, is summarized in Figure 2. Based on the user persona, a new web-based tool, Healthy Kai (Food) Checker—a searchable database of packaged food and drink products showing their traffic light classification—was identified as a key tool to support the New Zealand Policy implementation. Textbox 2 summarizes positive UX/UI attributes [17] we identified based on the review of similar available tools from Australia, Healthy Food Finder [36] in New South Wales, and FoodChecker [35] in Victoria, as well as a Canadian tool, Brand Name Food List [37], available in British Columbia. The function and feature requirements of the Healthy Kai Checker prioritized with the MoSCoW framework as “Must have” and “Should have” are indicated in Textbox 3.

**Figure 3:**
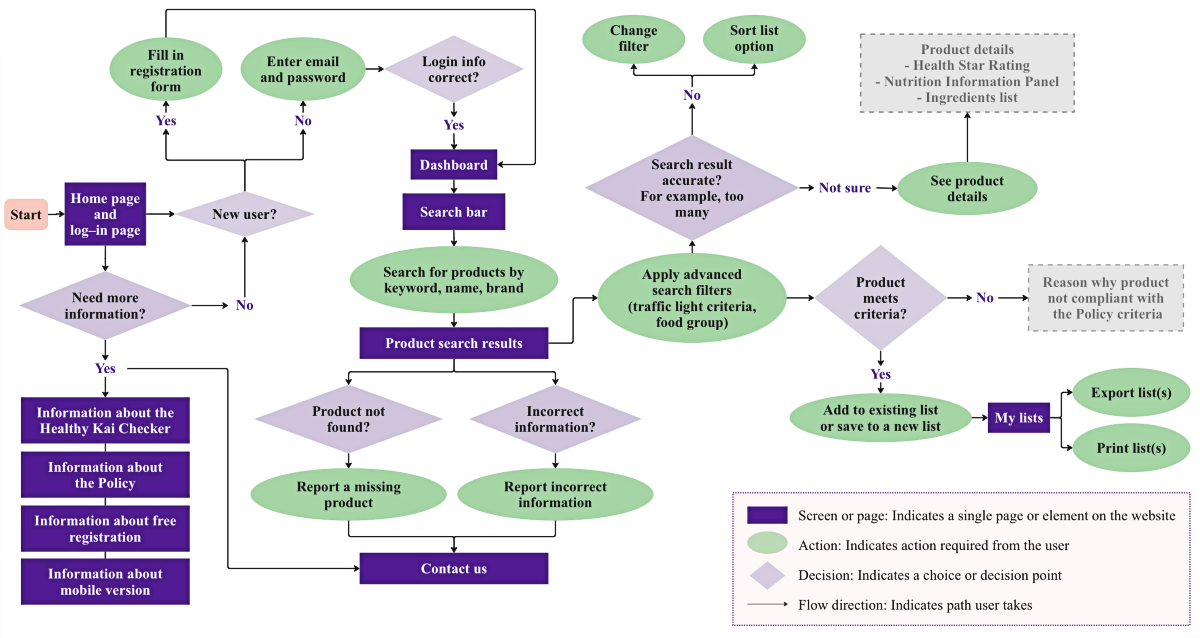
User flow diagram of the Healthy Kai (Food) Checker web-based tool. This diagram illustrates the user journey through the tool, detailing steps from initial log-in to final product selection. The user flow is designed to support users in implementing the National Healthy Food and Drink Policy in New Zealand hospitals. The green ovals show actions taken by the users, while the light purple diamonds indicate points where users choose their next action. Grayed-out rectangles show features classified as “Could have” or “Won’t have” according to the Must have, Should have, Could have, Won’t have (MoSCoW) prioritization framework, reflecting their lower priority and exclusion from the initial prototype tool.

The Healthy Kai Checker user flow (Figure 3) and functionality design included elements closely related to the “Must have” and “Should have” features outlined in Textbox 3 [38], for example, registration to create a free account, personalization to apply search filters and create lists of favorite products, educational content to explain terms and provide tips on how to use the tool, and a feedback mechanism to report an issue or suggest improvements. The corresponding Healthy Kai Checker site map is available in Multimedia Appendix 3.

**Favorable user experience and user interface features of web-based tools with product databases based on the review and comparison of similar existing web-based tools [17] in Australia [35,36] and Canada [37].**

**First impressions**

- Visually appealing (clear, fresh, and crisp look) and clean design
- Good use of white space for a less compact layout
- Colorful
- Modern feel

**Website visual design**

- Consistent visual style
- Consistent and clear branding
- Clear font
- Simple design
- Good color scheme and use of images
- A good indication of clickable elements

**Website interaction**

- Automatic suggestion of products in the search bar (predictive text)
- A clear indication of product compliance/traffic light classification
- Results displayed based on relevance to the search term (eg, for misspelled words)
- Product information in separate columns (name, brand, and size)
- Good column sorting function (both directions)
- Relevant search filters
- Explanation of why the product does not meet the Policy criteria
- Option to report missing products
- Ability to create own product lists
- Ability to return to previous screens without loss of content
- Confirmation of completed action
- Intuitive navigation

**Features and functions of the Healthy Kai (Food) Checker web-based tool prioritized with the Must have, Should have, Could have, Won’t have (MoSCoW) framework [38]. “Must have” features and functions were considered essential and critical, “Should have” were important but not critical, “Could have” were desirable but not critical, and “Won’t have” were out of scope and excluded in the current design but could be considered in the future. “Must have” and “Should have” were core elements to be incorporated into the new prototype web-based tool to support implementation of the National Healthy Food and Drink Policy in New Zealand hospitals.**

**Must have**

- A searchable list of packaged products (including *Red* products so that end users can quickly identify items that do not comply with the Policy instead of spending time classifying them manually).
- Ability to search by name, keyword, and brand.
- Basic and advanced search options (traffic light criteria and food group filters).
- A clear indication of the traffic light classification (text and color).
- Up-to-date product database (a database of products was created during the food and drink audits conducted as part of the HYPE study [14] that can be integrated).
- Engaging information about the Policy and Healthy Kai Checker.

**Should have**

- Product information separated into columns (name, size, and brand).
- Sort results function (alphabetically, product size, and traffic light classification).
- Ability to report a missing or incorrect products.
- Free registration requirement to view all products and unlock full access.
- Free registration requirement to create, save, export, and print product lists.
- No paid registration (free for all users).
- Mobile responsiveness for different devices.

**Could have**

- Detailed information about each product (eg, nutritional, ingredient list, and allergens)
- Clear product photographs.
- Filters related to allergens, suitability for vending machines.
- Explanation or indication of why a product does not meet the *Green* criteria.

**Won’t have**

- Self-assessment of products and recipes against the Policy criteria.
- Integration of healthy food and drink policies for New Zealand schools and early childhood education centers, or government-funded healthy school lunches program.
- Web-based Policy-relevant training modules.

### Develop Phase

**Figure 4:**
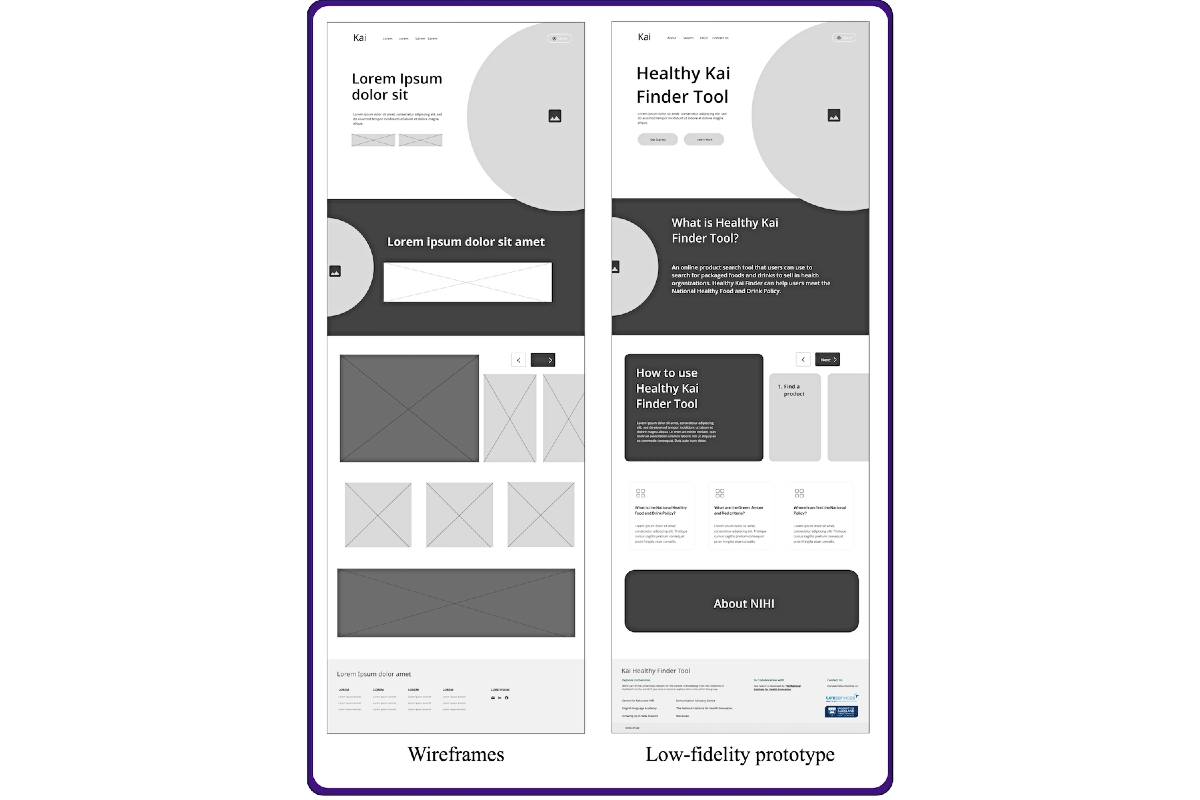
Wireframes and low-fidelity prototype of the Healthy Kai (Food) Checker web-based tool home page. This figure represents the initial design concepts for the tool, outlining the layout and structure of the home page and showcasing key elements of the interface design and basic functionality. The feedback from 11 public health nutrition professionals on the low-fidelity prototype was used to refine the web-based tool to support implementation of the National Healthy Food and Drink Policy in New Zealand hospitals.

The initial wireframe outline and the low-fidelity prototype for the home page are shown in Figure 4. The low-fidelity prototypes of the 2 design options for the product search and result dashboard are available in Multimedia Appendix 1. Feedback from the 11 Network members and the research team favored the option where the search box and result filters are at the top rather than on the side of the search page.

### Deliver Phase (Usability Testing)

Twelve individuals were invited to take part in the interactive interviews, of whom 1 declined due to lack of time, 2 were no longer employed in the same role, and 6 did not respond after 3 invitation emails. The final interview sample included 2 Network members (public health nutrition professionals) and 1 stakeholder who used the Policy in public health research on food environments. The interviews took an average of 49 (range 40-59) minutes to complete.

Participants generally found the Healthy Kai Checker prototype easy to use and logical to navigate. They appreciated the hyperlinks in the prototype that directed users to external web pages, such as the Ministry of Health website where the Policy was published. There was also an appreciation that the different pages within the tool could be reached through multiple buttons or links (ie, having multiple paths to 1 page), and participants provided additional suggestions on where specific buttons connecting to the various web pages of the tool or external links should be incorporated. All participants liked the color scheme and the purple and green palette, which made the tool look vibrant and fresh and differentiated it from other public health or government websites that typically use more muted colors. One suggestion was to ensure that images were representative of the diverse New Zealand population. The Healthy Kai Checker was positively perceived due to its simple and concise interface, which did not overwhelm users with too much information.

On the home page, participants suggested including more information about the free website registration and its benefits and placing that component closer to the top of the web page. The section informing users about the ability to use the tool on mobile or tablet devices was positively perceived and thought to be necessary in the current technology-driven working environments. However, a feature in the middle of the home page, “How to use the Healthy Kai Product Database” that contained a clickable carousel of moving parts from right to left, was deemed confusing. Participants suggested reducing the steps showing information on how to use the tool from 4 to 3 and displaying all of them at once on the main page, thus improving usability by eliminating the need to click on the various components to see the entire content.

On the product search and results page and the pages showing products saved in the user’s individual lists, the indication of products’ categorization as *Green*, *Amber,* or *Red* was perceived as unambiguous. The favorable features were the ability to apply advanced search filters (traffic light classification and the Policy’s food group) to display only those products with the specified characteristics. Three suggestions for improvements were incorporating an indication of the number of products in the search results and personalized product lists, making the “My Lists” feature more prominent, and making some of the buttons and writing bigger to improve visibility. Participants also thought that allowing manufacturers and suppliers to submit their products for classification and inclusion in the database, along with product photographs, was a good idea, and having a “Contact Us” page on the website that clearly outlined the steps required would facilitate this.

**Figure 5:**
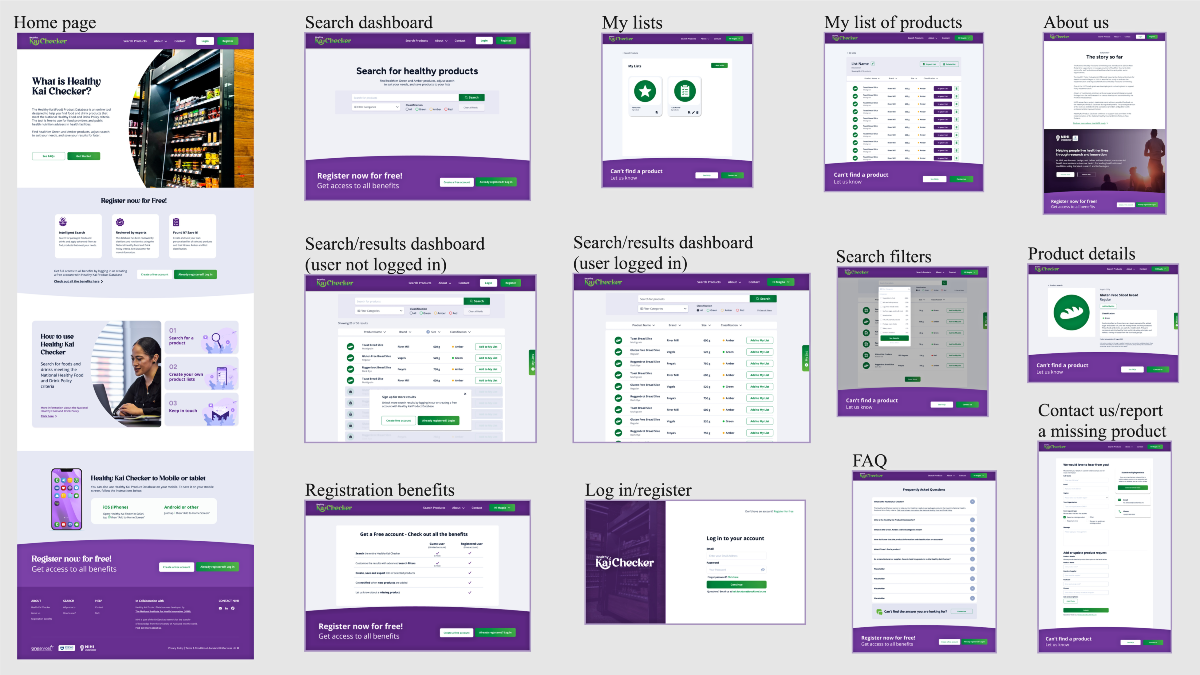
Screenshots of the various pages of the Healthy Kai (Food) Checker prototype web-based tool designed to support implementation of the National Healthy Food and Drink Policy in New Zealand hospitals. The tool is a searchable database of packaged food and drink products that classifies items according to the Policy’s nutritional criteria, with functions such as basic and advanced searches for products, sorting list options, the ability to compile a list of selected products, a means to report products missing from the database, and responsiveness for usage on different devices. FAQ: frequently asked questions.

For the remaining pages, participants suggested some frequently asked questions that users would likely be interested in, since the Network members had experience working closely with food providers to implement the Policy. Furthermore, minor aesthetic adjustments, such as reducing plain text and adding graphical elements or photographs, were suggested to make the pages more visually appealing. Screenshots of the most recent version of the Healthy Kai Checker prototype tool are shown in Figure 5.

## Discussion

### Principal Findings

We aimed to develop and test a new evidence-based, web-based prototype tool to support the adoption and implementation of the National Healthy Food and Drink Policy in New Zealand. The new Healthy Kai Checker web-based tool will reduce the burden on individual food providers and Network members associated with identifying and classifying products according to the Policy’s traffic light nutrition criteria. Until now, individual food providers and organizations had to undertake this essential implementation task manually, which is a repetitive and resource-intensive process.

The choice of a web-based tool in this study was driven by the increasingly digital or web-based practices and systems [45] that already saturate the grocery and retail market [22] and are increasingly used for individual behavioral change approaches [46] and research on food supply [47] and physical activity [24]. The scarcity of web-based or digital tools in New Zealand [17] for quickly checking the classification of packaged food and drink products represents an opportunity for the new Healthy Kai Checker to fill this significant gap and facilitate implementation of the Policy. Healthy Kai Checker was designed as an intuitive and logical web-based tool suitable for end users who may have limited nutrition knowledge and experience, and understanding of technical nutrition jargon, which can be overwhelming and complex [48-50]. Furthermore, the aim was to optimize user paths, accommodate varying levels of technological proficiency, and minimize the steps end users had to take [39] to search and identify suitable products.

The design and development of the Healthy Kai Checker followed the rigorous but flexible Double Diamond model [30], was guided by key UX/UI design principles [28,29,34], and was based on the comprehensive research our team conducted as part of the HYPE study [14,15,17,20]. While other design and development strategies could have resulted in a different or better tool, we are unable to make a direct comparison since the design and development process of similar tools in Australia [35,36] and Canada [37] had not been outlined in the literature. However, our approach provided key advantages of emphasizing and understanding user needs and stakeholder engagement, mitigating potential risks and challenges early in the process, and allowing iteration and creativity, leading to a unique, tailored, and effective solution to the identified resource gap [30,31].

### Implementation Considerations

To ensure a seamless user experience for all intended end users, Healthy Kai Checker would need to be further tested with food providers in New Zealand hospitals. Furthermore, expanding the current features to include other healthy food policies in New Zealand, such as the government-funded healthy school lunches program policy (Ka Ora, Ka Ako) [51], would require user testing with food providers working in school environments. However, it should be noted that usability testing and the incorporation of end user feedback are an ongoing process in the evolving digital space and a critical component of the product’s road map [30]. After Healthy Kai Checker is developed and released as a fully functioning web-based tool, it would need to be periodically updated to ensure that it maintains user-centered design and continues to offer advantages over other potential tools in the market.

Another key aspect of the Healthy Kai Checker is the ongoing management and maintenance of the food and drink product database that underpins the tool, as well as new updates of the algorithm embedded into the tool to facilitate the classification of products as *Green*, *Amber*, or *Red* [21]. The current database contains information on food and drinks collected during food availability audits in New Zealand hospitals as part of the HYPE study [14]. However, these data were captured in 2021/2022, and it is likely that, at some point, they will no longer accurately represent the supply on the market. Further manual field data collection, as used for the HYPE study, is likely too resource-intensive and costly to do regularly. Reaching out to the major New Zealand suppliers to encourage them to provide nutritional information regularly on their products could be a cost-effective strategy to streamline data collection and maintain the accuracy and currency of the dataset.

Another avenue includes obtaining product information directly from Healthy Kai Checker end users, as seen with crowdsourcing of supermarket data from the FoodSwitch [47] app users in Australia. The Healthy Kai Checker includes a feature where users can directly provide the necessary information and photographs about a missing or incorrect product for evaluation. An additional emerging method of data collection is the “web harvesting” or “data mining” approach used to automatically extract information from websites, such as web-based supermarket stores [52], and could also be explored to source data for the Healthy Kai Checker.

Regardless of the source of product data, classifying individual products into *Green*, *Amber*, and *Red* categories using the Policy’s criteria can be complex because some of the required information is not provided on the food packaging. The challenging classification of some products with the Policy criteria was observed during food and drink availability audits as part of the HYPE study [14], although a robust algorithm was developed and included in the web-based audit tool to make the process systematic [21]. Simplifying the Policy’s criteria could increase food provider and supplier understanding of the guidelines and streamline data collection to keep the product database up-to-date. Importantly, a classification algorithm would need to be revised if the Policy undergoes an update of its nutritional criteria, representing an additional cost associated with ongoing maintenance of the Healthy Kai Checker.

### Strengths and Limitations

There are several strengths in this study. First, the design and development of the Healthy Kai Checker were informed by comprehensive research findings and aligned with the needs of the Policy implementers. Second, the UX/UI designer used the latest website design techniques and best practices to ensure the user-friendliness of the tool. Third, feedback and suggestions provided by stakeholders and participants were incorporated into the tool to improve its usability. However, there were some limitations. Although the Healthy Kai Checker prototype has been tested with 1 group of potential end users, testing has not yet been conducted with the New Zealand food providers because they did not respond to the invitation to take part in the research. Future research should explore the usability of the prototype tool with food providers and stakeholders associated with other policies or settings. User testing should also be conducted once the Healthy Kai Checker is a fully functioning website. Since the tool is still a prototype, this study did not explore the effectiveness and impact of the Healthy Kai Checker as a tool in the Policy implementation. Research on the effectiveness of tools and resources to support healthy food policy implementation is generally lacking, and future studies should focus on evaluating the effectiveness of tools in this space.

### Conclusions

This study comprehensively designed and developed a new web-based Healthy Kai Checker tool, a searchable database of packaged food and drink products, to facilitate the implementation of the National Healthy Food and Drink Policy in New Zealand. The development of the tool was guided by the Double Diamond model and UX/UI design principles. Feedback received from project stakeholders was incorporated into the prototype, and comprehensive user-testing results further improved the design and usability of the prototype tool. The Healthy Kai Checker could provide valuable support to New Zealand hospital food providers and others who support implementation of the Policy.

## Appendix

Multimedia Appendix 1 Low-fidelity prototype feedback document.

Multimedia Appendix 2 Usability testing of the Healthy Kai Checker interactive interview guide.

Multimedia Appendix 3 Healthy Kai Checker site map.

## Abbreviations

DHB: district health board
HYPE: Healthy Policy Evaluation
MoSCoW: Must have, Should have, Could have, Won’t have
UX/UI: user experience and user interface

## Data Availability

The interview data analyzed in this study are not publicly available due to presence of sensitive and personally identifiable qualitative data that could compromise the anonymity of the participants. The remaining datasets generated and analyzed during this study are available from the corresponding author on reasonable request.
