# Supplementary material for: Healthy Kai (Food) Checker Web-Based Tool to Support Healthy Food Policy Implementation: Development and Usability Study
Source: JMIR Form Res. 2025 Jan 13;9:e60447. doi: 10.2196/60447 (PMC11773278; doi:10.2196/60447)
Supplement: Multimedia Appendix 1 [file formative_v9i1e60447_app1.pdf]

Low-fidelity prototype feedback document

This document contains a low-fidelity prototype of the tool that will be a web-based app. The tool has been developed with a UX/UI (user experience) designer. It is based on the results from the international systematic literature review, a review of tools and resources in New Zealand, Australia and Canada, and interviews with New Zealand stakeholders. The tool's primary focus at this stage will be a searchable database of compliant healthy food and drink products. To better understand how we can improve the prototype and make the tool highly useful to the end users (e.g., food providers, vending machine suppliers and those supporting policy implementation), we would appreciate your feedback and advice as important stakeholders of this mahi (work). Below are example screenshots and some questions we are primarily interested in at this stage, but please feel free to share any other feedback you might have.

General structure & Homepage

1. What do you think of the navigation structure?
2. What do you like or dislike?
3. What other feature or information would you like to see on the web app?
4. What do you think of the Homepage structure?
5. Thinking about the homepage, what high-level information will keep users interested and use the search function of the web app?

Dashboard navigation & features

Option 1 (Advanced search on the left bar) or Option 2 (Search filters displayed on the top with multiple selections)

1. What navigation option for dashboard searching do you prefer?
2. Why do you prefer this option?
3. What do you think about the list feature?
4. What additional options or features do you think are needed to make it easy to use?
5. Do you have any feedback for us to improve the web-app?

General structure & Homepage

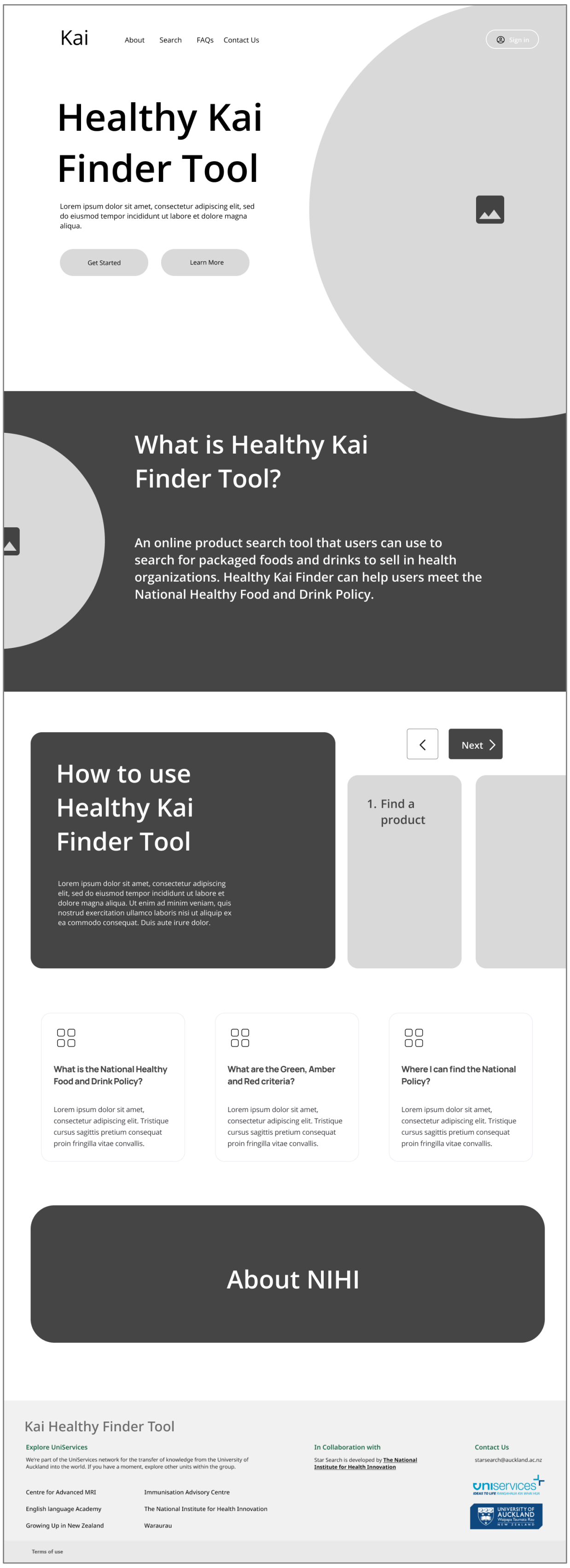

Dashboard navigation & features

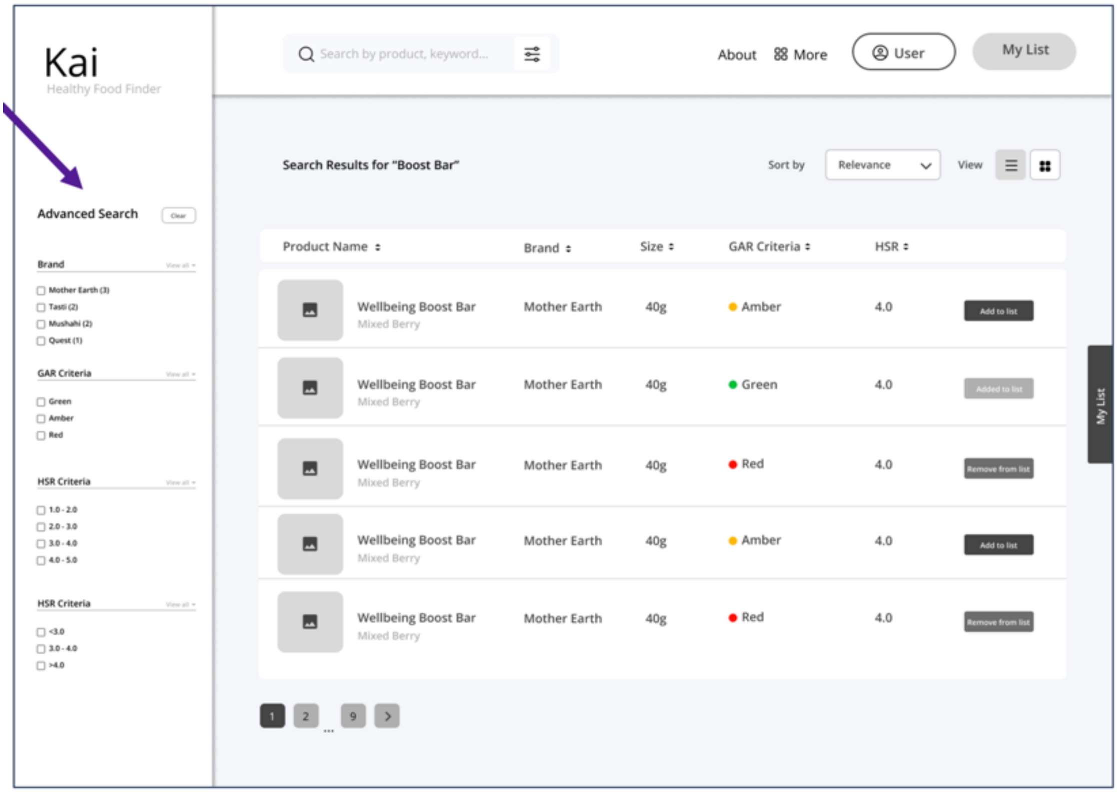

Low-fidelity prototype, Option 1, search and filter options on the left (indicated with an arrow)

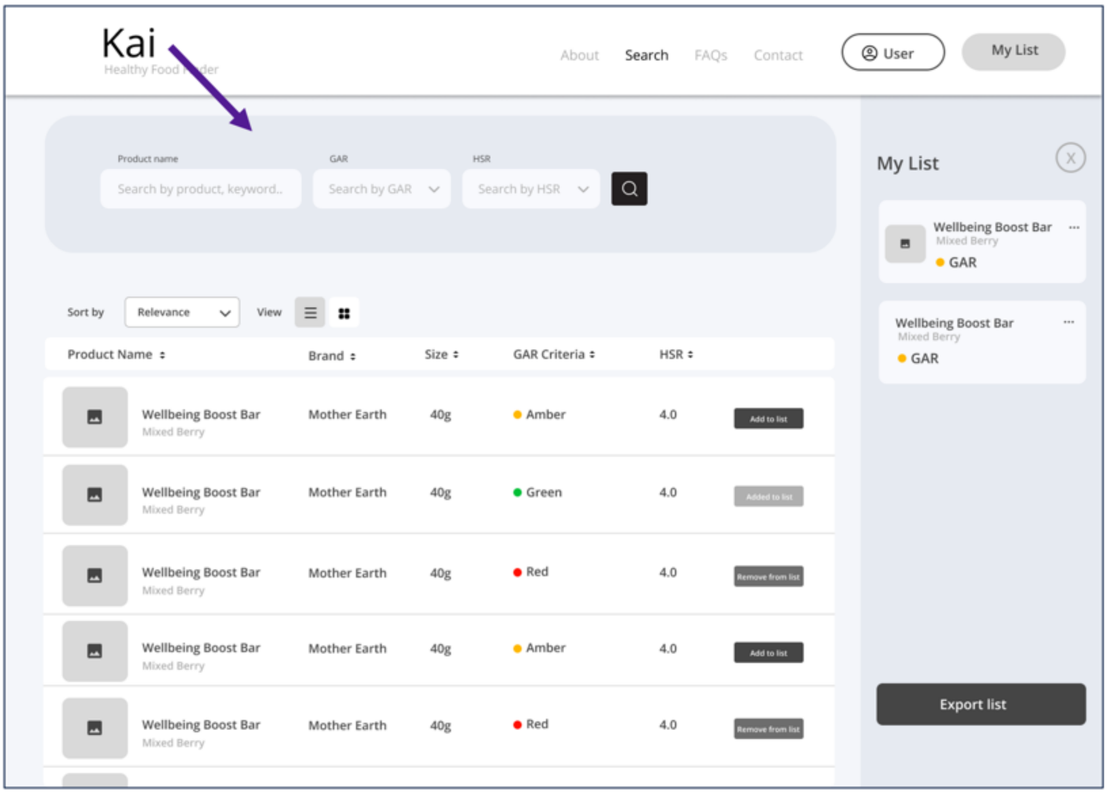

Low-fidelity prototype, Option 2, search and filter options on the top (indicated with an arrow)
