# Supplementary material for: Healthy Kai (Food) Checker Web-Based Tool to Support Healthy Food Policy Implementation: Development and Usability Study
Source: JMIR Form Res. 2025 Jan 13;9:e60447. doi: 10.2196/60447 (PMC11773278; doi:10.2196/60447)
Supplement: Multimedia Appendix 2 [file formative_v9i1e60447_app2.pdf]

Usability testing of the Healthy Kai Checker interactive interview guide

Tasks / Questions	Click Path	Observations	Quotes	Task Completion
	Record what path the participant took to complete the task.	Note down behaviours, opinions, and attitudes along with any errors, issues, or areas of confusion.	Note any significant quotes	Choose if the task was:<br>1 - easy to complete<br>2 - completed but with difficulty<br>3 - not completed
Scenario 1:<br>**If I said, "This tool is a database of healthy-compliant products in line with the National Healthy Food and Drink Policy for hospitals and other public sector workplaces in New Zealand," what do you think you might use this tool for?**				
Scenario 2:<br>**As a first time user, or a returning user, you are trying to find some more information about Healthy Kai Finder on the Homepage**				
Task 2a:<br>Find the main information about Healthy Kai Finder on the Homepage.				
Question 2a:<br>What do you think of the Homepage structure?				
Question 2b:<br>What other features or information would you like to see on the web app?				
Question 2c:<br>Thinking about the homepage, what high-level information will keep users interested and use the search function of the web app?				
Question 2d:<br>How can the Homepage be improved?				
Scenario 3:<br>**We are staying on the homepage. Imagine you would like to search for a specific product in the database**				

Tasks / Questions	Click Path	Observations	Quotes	Task Completion
Task 3a:<br>Get started with searching for a product. How would you go about doing that?				
Task 3b:<br>Let's say you now wanted to search for "[Product name example]" How would you do that?				
Scenario 4:<br>**Now that a list of products appears, you want to organise and narrow down your search results in a specific way**				
Task 4a:<br>Let's say you want to filter the products by *Green*, *Amber*, and *Red* criteria. How would you do that?				
Task 4b:<br>Let's say you now wanted to narrow the search results. How would you do that?				
Question 4a:<br>What narrowing criteria would be most interesting to sort the products? Would having the HSR (Health Star Rating) listed be helpful?				
Question 4b:<br>What do you think of the dashboard navigation structure?				
Question 4c:<br>What do you like or dislike?				
Question 4d:<br>What other features or information would you like to see on the navigation dashboard?				
Question 4e:<br>How engaging is the dashboard? How can we make it more engaging?				

Tasks / Questions	Click Path	Observations	Quotes	Task Completion
Question 4f:<br>What information will keep users interested and use the search function of the web app?				
Question 4g:<br>How can the search dashboard be improved?				
Question 4h:<br>Would you like product photos to be included in the product list? How important is it?				
Scenario 5:<br>**Now that you have found product(s) you were looking for, you would like to save these specific products for later use so you can come back and access it later**				
Task 5a:<br>Try to add a product to "My List" and then try to remove it.				
Question 5a:<br>What do you like or dislike about "My List" feature?				
FU Question 1:<br>How did you feel about the Healthy Kai Finder Tool overall?<br>What did you like about it?<br>What did you dislike about it?				
FU Question 2:<br>As a user of this tool, what additional options, features, or information would you like to have on Healthy Kai Finder Tool to make it more useful for you?				
FU Question 3:<br>Healthy Kai Finder will be a web app suited for use on the laptop/computer screen. How important would a mobile version of the app be to you?<br>Would you use it more on the laptop/computer or a mobile phone?				
