# Supplementary figures and images for: Healthy Kai (Food) Checker Web-Based Tool to Support Healthy Food Policy Implementation: Development and Usability Study
Source: JMIR Form Res. 2025 Jan 13;9:e60447. doi: 10.2196/60447 (PMC11773278; doi:10.2196/60447)

## Healthy Kai Checker Site Map

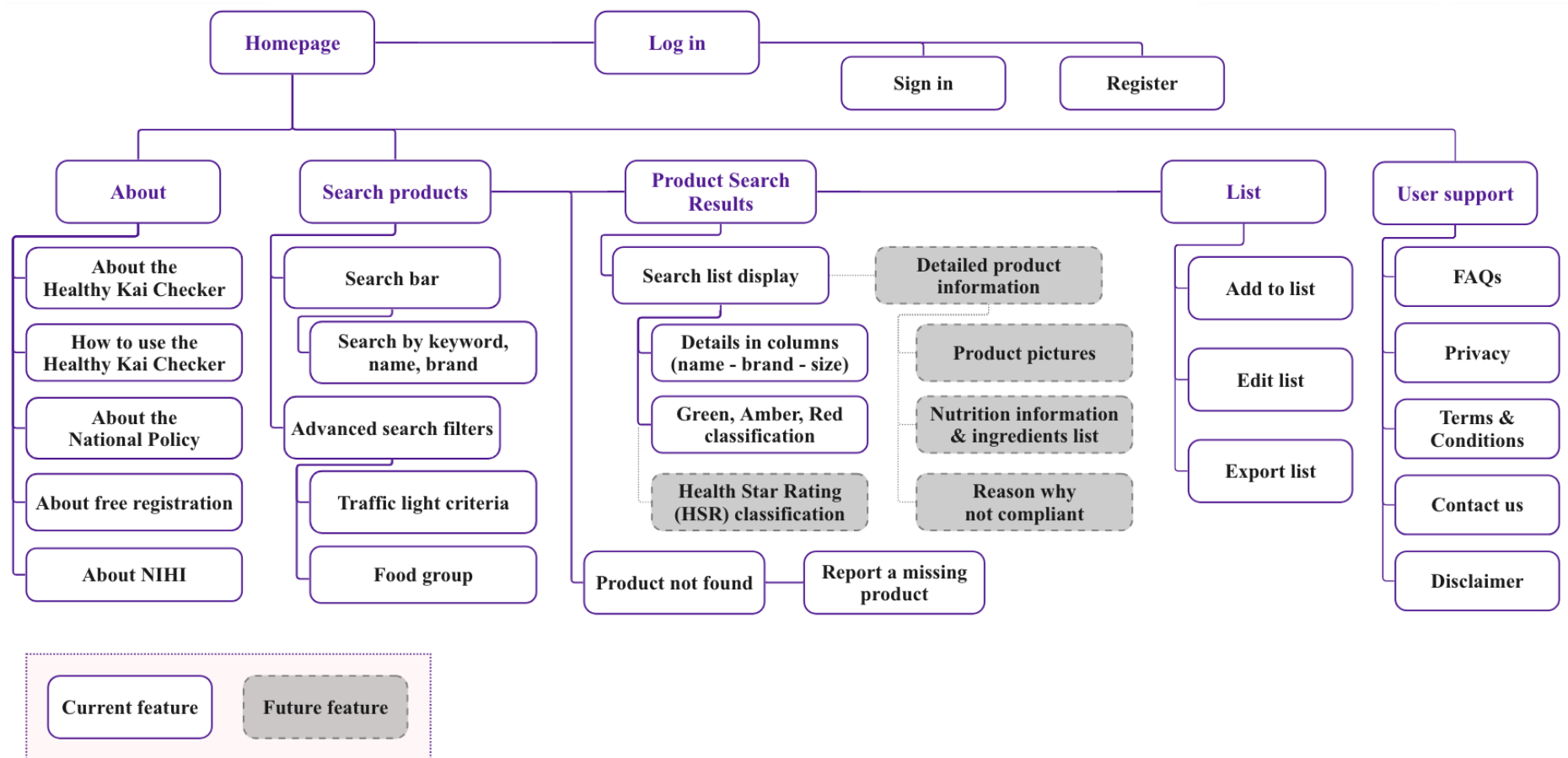

Supplement: Multimedia Appendix 3 [file formative_v9i1e60447_app3.pdf]
